# *Candida albicans* Osteomyelitis Pubis: The Possible Pathogenic Role of Pelvic Irradiation

**DOI:** 10.1155/2017/5961917

**Published:** 2017-12-03

**Authors:** Cristiana Iacuzzo, Jacopo Monticelli

**Affiliations:** ^1^Department of General Surgery, University Hospital of Trieste, “Ospedale di Cattinara”, Strada di Fiume 447, 34149 Trieste, Italy; ^2^Infectious Diseases Unit, University Hospital of Trieste, “Ospedale Maggiore”, Piazza dell'Ospitale 2, 34125 Trieste, Italy

## Abstract

Radiation to the pelvis, mainly directed against either prostatic or gynecologic cancers, is burdened by a lot of complications. The genitourinary tract is most frequently involved, presenting with bladder irritation, incontinence, and fertility disorders. However, side effects of radiation can also affect the bone, usually causing an osteolytic process which deteriorates the bone structure and leads to fractures, avascular necrosis, and other pathological insults. Here, we describe a case of *Candida albicans* osteomyelitis of the pubic symphysis as late complication of pelvic radiotherapy performed against prostate cancer.

## 1. Case Presentation

**Figure 1:**
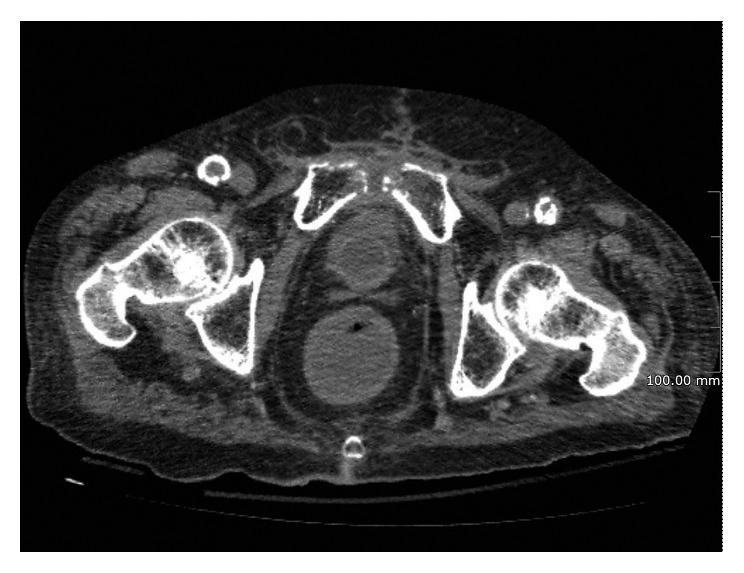
CT scan transverse section showing the erosion of the pubic symphysis surrounded by inflammatory tissue.

An 86-year-old Caucasian male presented to our department complaining bilateral thigh pain and ambulatory disability. His only comorbidity was arterial hypertension, successfully treated with ACE inhibitors. He lacked any history of traumas. In 2005, the patient had developed a prostate cancer (PC) treated by radical prostatectomy and adjuvant radiotherapy without early complications. Ten years later, the patient came to our attention having already performed a computerized tomography (CT) scan at another health facility. The CT scan demonstrated an erosion of the pubic symphysis (Figure 1) associated with an inflammatory collection within the pelvic cavity, extending throughout the femoral triangles and within the layers of the abductor muscles of the thighs bilaterally. Lab tests were not significantly altered, with serum leukocytes of 9,030 cells/mm^3^ and C-reactive protein (CRP) of 11.9 mg/L.

The abscess of the left limb appeared to be small, deep and close to the femoral vessels, thus rendering its drainage dangerous, and in our opinion potentially counterproductive. Therefore, we decided to perform a surgical drainage of the sole right limb, whereas to treat the left conservatively at first. The drainage of the right abscess revealed an opalescent fluid in which various colonies of a pan-sensitive *Candida albicans* were isolated (fluconazole minimum inhibitory concentration: 0.5 mg/L). Therefore, the previously empirical antibiotic therapy with intravenous (IV) imipenem was stopped, and the patient was started on IV fluconazole (800 mg as loading dose and then 400 mg daily).

**Figure 2:**
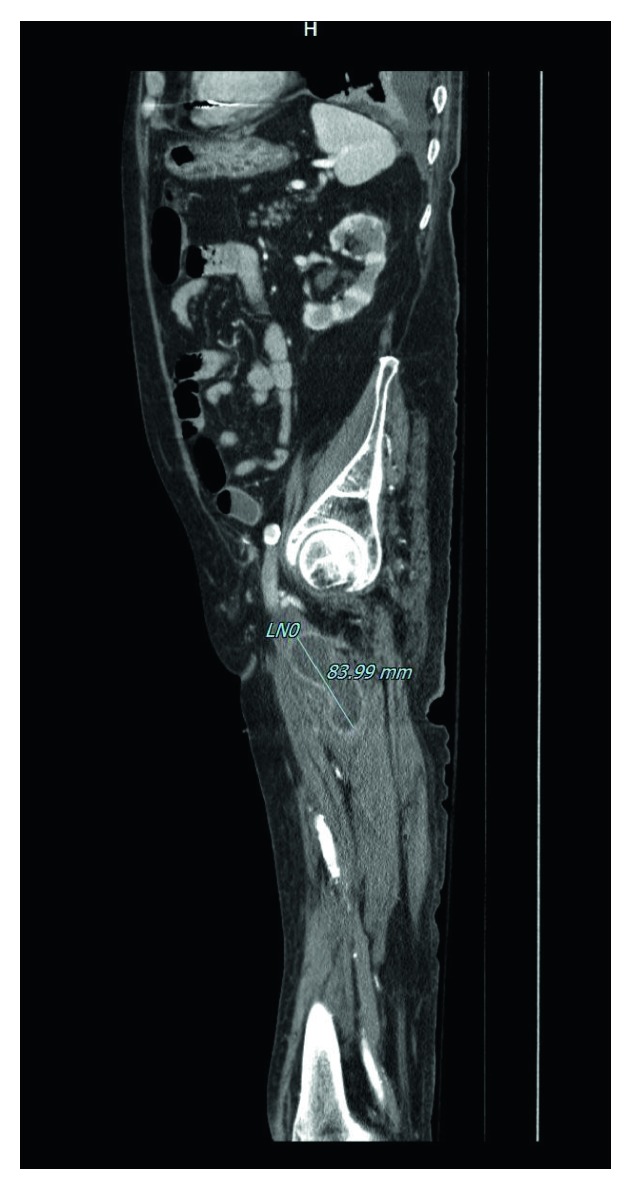
CT scan sagittal section showing the collection still present in the left thigh of the patient after the surgical procedure on the right side.

**Figure 3:**
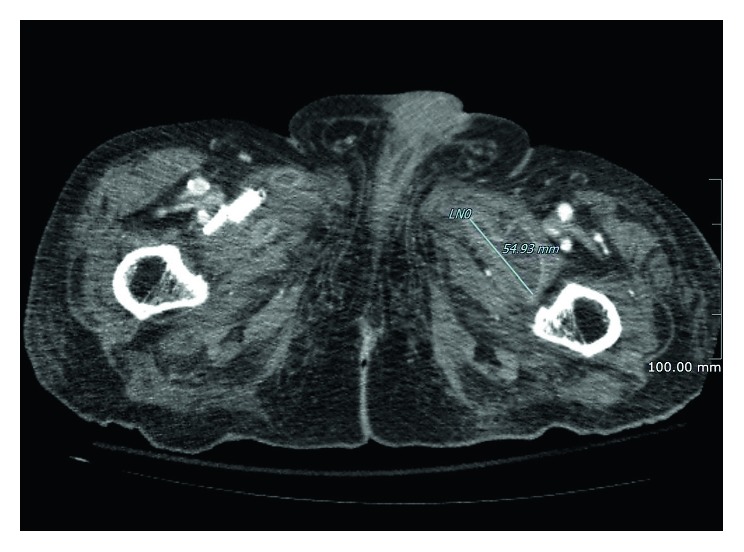
Transverse section of the same CT scan showing the left collection yet a drastic dimensional reduction of the right collection.

The early postoperative course was complicated by local infection of the surgical wound. *Pseudomonas aeruginosa* was isolated from a wound tissue sample, thus leading to the decision of starting adequate IV antibiotic therapy with ciprofloxacin and ceftazidime. On the 10th postoperative day (POD), a CT scan was performed to check the evolution of the local situation. The radiological images showed that the abscess was almost completely regressed in the right thigh, whereas, albeit slightly reduced, it was still present on the left side (Figures 2 and 3).

At this point, a US-guided needle aspiration of the residual collection was undertaken, and the same strain of *Candida albicans* was isolated. Therefore, the patient continued to be treated with IV fluconazole for 15 days and then shifted to oral fluconazole 400 mg daily. On the 20th POD, a scintigraphy with ^99m^TC-HMPAO-labeled autologous leukocytes combined with CT (^99m^TC-HMPAO-SPECT/CT) was performed. Since the exam demonstrated the regression of the pelvic osteomyelitis and inflammatory markers were within the normal range on lab tests, the patient was discharged with oral fluconazole and an adequate physical rehabilitation program in order to regain his complete walking ability.

## 2. Discussion

Radiotherapy is nowadays a potentially curative technique against radiosensitive tumors like PC, and this justifies its large use in clinical practice. However, its complications are well known too, and that is why in the last decades, many efforts have been made to escalate radiation doses whilst maintaining the same efficacy against PC [1, 2]. The degree and extension of radiation-induced side effects in normal tissues in fact depend mostly on treatment-related factors—such as total dose, dose per fraction, fractionation schedule, total treatment time, irradiated volume, and type of radiation. Patient-related factors (e.g., age, comorbidity, and genetic radiation susceptibility), as well as additional treatment modalities, may worsen the normal tissue injury. While early radiation effects may be transient, late effects tend to be irreversible and may even be progressive.

Irradiation leads to acute vascular changes within 24 hours. Oxygen radicals act directly on DNA, resulting in tissue injury, which includes endothelial cell damage, increased permeability, edema, and fibrin accumulation. An inflammatory response comprehensive of macrophage activation, release of cytokines, and increased oxygen consumption leads to vascular injury and hypoxia. Activation of cytokines leads to fibrosis and impaired function in blood and lymph vessels. Additional hypoxia determines chronic radiation-induced injury. Abnormal microenvironmental conditions exist even after radiation treatment is over and will perpetuate the tissue damage [3].

In the last decades, dose escalation has been possible, thanks to the development of software and devices built to avoid dose delivery to the surrounding healthy tissue. Dose escalation in external beam radiotherapy (EBRT) has been shown to improve regional control, disease-free survival, distant disease–free survival, mortality, and overall survival in intermediate- and high-risk PC [4] beyond reducing toxicity. Brachytherapy (BT) permits an extreme dose escalation, far exceeding other techniques. Intensity-modulated radiotherapy (IMRT) is a technique that allows to better shape the high-dose region on the target. Volumetric-modulated arc therapy (VMAT) is an advanced form of IMRT that improves target volume coverage and spares healthy tissue [5]. The last RT techniques utilize heavy particles—like protons and carbon ions—that seem to have maximum effect on the PC while minimizing the impact on the surrounding healthy tissue [6].

Regardless of these unequivocal improvements, radiation-induced osteitis is a well-described phenomenon consisting of reduction in bone vasculature as a consequence of endarteritis and periarteritis. Swelling and vacuolization of the endothelial cells lead to a loss of vascularization, thus resulting in the development of sclerotic connective tissue and fibrosis. Besides the damages to the vasculature, radiation-induced increase in osteoclast activity, together with reduction in osteoblasts number and function, results in bone reabsorption and atrophy, therefore impairing bone mineralization and reducing the production of mature bone tissue. This exposes the bone to all kinds of pathological insults, which may lead, for example, to osteomyelitis [7].

Our patient had undergone a conventionally fractionated IMRT dose schedule (64 Gy/32 fractions/6.5 weeks). The clinical target volume (CTV) had started 6 mm below the anastomosis, had extended to cover the prostate bed 3 cm superiorly along the posterior side of the pubic symphysis, and had covered the space posterior to the bladder. Although the site of the osteomyelitis had not been part of the elective area, the dose volume histogram had shown 61% of the pubic bone receiving 30 Gy, 46% receiving 40 Gy, and 9% receiving 50 Gy.

Although the acute and late toxicity associated with radical RT has been well described, the toxicity of adjuvant RT has been less well characterized but seems to be substantially lower. Besides, if we consider that inadequate CTV coverage has been identified as an important potential cause of local relapse [8], we believe that stricter dose constraints may result dangerous and counterproductive.

Few authors described osteomyelitis pubis as a complication of pelvic radiotherapy, for bladder, rectal, and prostatic cancers, and these cases are often, if not always, associated with fistulas as an additional and main source of infection [9, 10]. Other authors described osteomyelitis pubis in prostate cancer survivors who had undergone alternatively radiation alone or surgery and salvage radiation [7]. This may suggest a possible adding role of surgery in the development of the infectious complication.

Most reports of *Candida* osteomyelitis are limited to individual descriptions [11] and relatively small case series. The pathogenesis of the infection is not still perfectly understood: the apparent mechanisms consist in haematogenous dissemination, direct inoculation, and contiguous infection. The pubic bone is an unusual site for the already rare infection, which usually affects the vertebrae, femora, ribs, sternum, and humeri. In Gamaletsou et al. [12] series, median age for *Candida* osteomyelitis was 30 years, with a predominance of males. The majority of patients were not significantly immunosuppressed (i.e., underlying hematology malignancy, transplantation, or solid tumor). Only a minority of patients had trauma or open wounds. In nearly one-half of patients, osteomyelitis was the first proven site of *Candida* involvement, where the remaining cases initially had candidemia or candidiasis (cutaneous and subcutaneous infection, urogenital infection, eye infection, abdominal infection, oral cavity infection, lymph node infection, pneumonia, mediastinitis, uterus infection, and hepatitis). Consistent with a predominantly haematogenous process of dissemination, the majority of patients had 2 or more sites of infection. Markers of inflammation were usually only minimally elevated. Concurrent bacterial infection was not uncommon (in particular, *Staphylococcus aureus*). This inevitably brings into question some of the classical risk factors for fungal infections (i.e., immunodeficiency, prolonged hospitalization, older age, comorbidities, need for parenteral nutrition, and history of trauma).

The most appropriate therapeutic approach for *Candida* osteomyelitis usually consists of antimicrobial therapy either alone or combined with a surgical approach in about half of the cases. The type of antimicrobial therapy depends on the pathogen species and sensitivity. Fluconazole is the most commonly administered agent, since it has less adverse events than amphotericin B and a more favorable bone penetration than echinocandins [13].

Both *Candida* osteomyelitis and pubic bone osteomyelitis are extremely rare, thus rendering pubic bone *Candida* osteomyelitis an exceptionally unusual condition. Therefore, its diagnosis represents a challenge for the physician, and the level of suspicion has to be high especially in immunocompromised patients as well as in those who have previously undergone RT. A microbiological examination of a deep sample of the infected zone is the best way to achieve a correct diagnosis. Although the therapeutic approach against *Candida* osteomyelitis is already defined and effective, the pathologic mechanisms and diffusion of this rare infection are far from being fully understood. Likewise, the relation between RT and late-onset *Candida albicans* pubic osteomyelitis is still to be proven.
